# Opening the “black box” of informed consent appointments for genome sequencing: a multisite observational study

**DOI:** 10.1038/s41436-018-0310-3

**Published:** 2018-10-01

**Authors:** Saskia C. Sanderson, Celine Lewis, Christine Patch, Melissa Hill, Maria Bitner-Glindzicz, Lyn S. Chitty

**Affiliations:** 1 North East Thames Regional Genetics Service, Great Ormond Street Hospital for Children NHS Foundation Trust, London, UK; 2 UCL Great Ormond Street Institute of Child Health, London, UK; 3 Florence Nightingale Faculty of Nursing and Midwifery, King’s College London, London, UK; 4 Genomics England, Queen Mary University of London, Dawson Hall, London, UK

**Keywords:** genome sequencing, next-generation sequencing, informed consent, education, communication skills

## Abstract

**Purpose:**

Little is known about how health-care professionals communicate with patients about consenting to genome sequencing. We therefore examined what topics health-care professionals covered and what questions patients asked during consent conversations.

**Methods:**

Twenty-one genome sequencing consent appointments were audio recorded and analyzed. Participants were 35 individuals being invited to participate in the 100,000 Genomes Project (14 participants with rare diseases, 21 relatives), and 10 health-care professionals (“consenters”).

**Results:**

Two-thirds of participants’ questions were substantive (e.g., genetics and inheritance); one-third administrative (e.g., filling in the consent form). Consenters usually (19/21) emphasized participant choice about secondary findings, but less often (13/21) emphasized the uncertainty about associated disease risks. Consenters primarily used passive statements and closed-ended, rather than open-ended, questions to invite participants’ questions and concerns. In two appointments, one parent expressed negative or uncertain views about secondary findings, but after discussion with the other parent opted to receive them.

**Conclusion:**

Health-care professionals need to be prepared to answer patients’ questions about genetics to facilitate genome sequencing consent. Health-care professionals’ education also needs to address how to effectively listen and elicit each patient’s questions and views, and how to discuss uncertainty around the disease risks associated with secondary findings.

## Introduction

Genome sequencing has the potential to transform much of health care: genetic diagnoses will potentially be reached far more quickly than ever before in patients with rare diseases of unknown cause, reducing “diagnostic odysseys,” and will eventually lead to improved personalized treatments and prevention.^1^ While there is great potential for genome sequencing to impact rare disease diagnoses and treatments, there are also practical and ethical challenges. One challenge is how to support informed consent. The decision for patients is not always straightforward because potential benefits, e.g., diagnosis, need to be weighed against other considerations, e.g., family implications and data security.^2^ Individuals also need to make informed choices regarding secondary findings, e.g., for disease-predisposing variants.^3^ The secondary findings issue is one of the most significant ethical challenges arising from clinical genome sequencing.^2,3^ Secondary findings decisions are particularly complex given their uncertainty, e.g., variable expressivity of variants in even well-studied genes such as *BRCA1* in individuals from unselected populations.^4^

As genome sequencing moves from research to clinical contexts, as with other investigations, individuals have the right to consent or not based on communication of understandable and adequate information.^5^ It is important to understand what approaches are most appropriate for research and diagnostic genome sequencing consent. Informed consent is an important cornerstone ensuring research involving human participants is ethical and responsible, with respect for participants’ autonomy.^6^ For participants to provide meaningful informed consent, current UK National Health Service (NHS) research guidelines emphasize participants should have in-person conversations with suitably trained researchers or health-care professionals and the opportunity to ask questions^7^ after receiving an information sheet, before signing a consent form.

The 100,000 Genomes Project provides a unique framework within which to begin to explore genome sequencing consent for research and clinical care. The genomes of NHS patients with rare undiagnosed diseases and cancers are being sequenced to improve diagnoses and treatments, and bring predicted benefits of genomic medicine to patients.^8^ It will also “mainstream” genomics by embedding next-generation sequencing (including genome sequencing) into the health-care system, including routine ordering of genomic tests by clinicians who are not clinical geneticists. In addition to practical challenges around implementing genome sequencing on a massive scale, there are numerous ethical and psychosocial challenges, particularly around informed consent.^9^ Although the 100,000 Genomes Project information sheets and consent forms are detailed and standardized, little is known about the consent conversations. Examining how consent appointments function in the 100,000 Genomes Project could shed light on health-care providers’ and participants’ communication around genome sequencing consent, and inform policy for integrating genome sequencing into health care.

A useful methodology to illuminate the “black box” of consent appointments is the qualitative analysis of appointment audio or video recordings.^10^ This can allow analysis of appointment structure and content and exploration of themes emerging during the discourse. Examining conversation content and identifying topics from the information sheet and/or consent form that are discussed less often may suggest topics that are challenging to discuss or systematically omitted for other reasons.

We therefore examined whether and how different types of information were communicated between consenters and participants by conducting qualitative analyses of audio-recorded consent appointments in the rare disease arm of the 100,000 Genomes Project. Our specific aims were to (1) describe the overarching structure of genome sequencing consent appointments, (2) describe the content of the genome sequencing information provided by consenters, (3) explore what questions potential participants ask about genome sequencing, and (4) explore the nature and content of consenter–participant communication around secondary findings. Our overall objective was to provide insights and guidance on how discussions about consenting to genome sequencing could be conducted when used in mainstream clinical care.

## Materials and methods

### Study design

This was an observational study involving the audio recording of in-person consent appointments between health-care professionals (“consenters”) and potential participants for the 100,000 Genomes Project. Approval for this study was obtained from the NHS Research Ethics Committee West Midlands (15/WM/0258).

### Setting

Recruitment was conducted through four London hospitals in two Genomic Medicine Centres between June 2016 and January 2017. In the 100,000 Genomes Project, participants receive genomic information relevant to their condition (“main finding”). Participants can also consent to “secondary findings” being looked for and reported. These are variants associated with serious, life-threatening conditions that can potentially be prevented, screened for, and/or treated.^11^ The genes in which variants will be looked for include those predisposing to cancer (e.g., *BRCA1/2*, Lynch syndrome), and familial hypercholesterolemia. Where both parents participate, the couple can opt to receive carrier information (cystic fibrosis).^11^ Women can opt to receive information about carrier status for X-linked diseases (Duchenne muscular dystrophy). (See Supplementary Material for a 100,000 Genomes Project consent form in use at the time of this study).

### Participants and recruitment

This study included consenters responsible for the consent process, and potential participants (patients with rare diseases plus their parents/relatives) being recruited to the 100,000 Genomes Project. Consenters have a range of backgrounds including genetic counseling, research nursing, and other postgraduate training. All consenters take the online training course Preparing for the Consent Conversation^12^ and receive face-to-face training. Prior to recruitment, potential participants are identified as eligible by their consultants, who may provide brief information before referring them to consenters for more detailed information and discussion.

Consenters were invited to take part in our study and asked whether they wished to consent. Of 12 consenters invited, 2 declined. To enroll participants, consenters approached patients prior to consenting them into the 100,000 Genomes Project, asked if they would be willing to have the consultation audio recorded, and gave them an information sheet about our study. Consenters approached participants in a continuous way so as not to bias the sample. A minimum of two consenters was recruited at each hospital; each was given an audio-recording device to record the conversations after participants had given written consent.

Participants were eligible if they were adult patients with rare diseases or parents/relatives eligible for the 100,000 Genomes Project including those who accepted, declined, or deferred the decision; had capacity; were able to read information materials; and were having the discussion in English. Of 22 eligible families invited to participate, 1 declined due to distress about their child’s condition. Recruitment continued until we achieved thematic saturation as indicated by data redundancy, i.e., when no new themes emerged.^13^

### Data analysis

We used an interpretative qualitative methodology.^14^ First, all audio recordings were transcribed verbatim by external transcribers, and transcripts were de-identified (e.g., participant names and hospital site were removed) before the researchers read them. The research team comprised three postdoctoral researchers (S.C.S., C.L., M.H.) with expertise in conducting qualitative analyses, and three genetics health-care providers (C.P., M.B.-G., L.S.C.) of whom two had experience supervising qualitative analyses. Directed content analysis^14,15^ was conducted to examine the overarching structure of appointments. Specifically, each transcript was coded and categorized independently by two investigators (S.C.S., C.L.) as being consenter-led or participant-led: transcripts categorized as primarily consenter-led were further examined for whether participants actively participated, i.e., whether they expressed their views and/or concerns. The two investigators then compared codes and disagreements were resolved. During this analysis phase, consenters’ questions were identified and coded as either “open-ended” or “closed-ended.” Thematic analysis was then used to analyze themes, which involved an iterative process where data were coded, compared, contrasted, and refined to generate emergent themes.^13^ To develop the codebook, one investigator (S.C.S.) read all transcripts and a second investigator (C.L.) independently read a subset; both developed draft codebooks based on reading and reviewing the same transcript; these were compared and combined into a single codebook after discussion. NVivo 10 (QSR International, Australia) was used to manage the data and facilitate coding. The two investigators independently coded a second transcript in NVivo; disagreements were resolved; minor codebook revisions were made. Each investigator independently coded a third transcript: the kappa was 0.78 indicating good interrater agreement; this codebook version was used subsequently. To ensure rigor and increase authenticity, remaining transcripts were coded by multiple investigators (S.C.S., C.L., M.H.) with varying levels of familiarity with genome sequencing, and emerging themes were checked at multiple timepoints with the genetics health-care providers and two patient representatives.

## Results

### Participants

There were 45 participants: 10 health-care professionals (consenters), and 35 potential participants in the 100,000 Genomes Project, in 21 consent appointments. All (10/10) consenters and 16/35 participants were female. See Table 1 for additional characteristics. In 20/21 appointments all potential participants consented to take part in the 100,000 Genomes Project; one adult patient deferred the decision.

**Table 1:** Consenter and participant characteristics

Characteristics	Number (*N*)
**Consenters (total** ***n*** ** = 10)**	
Gender	
Female	10
Male	0
Highest level of education/training	
No degree: A-levels	1
Undergraduate degree: BA	1
Postgraduate degree: MSc	5
Doctorate degree: PhD	1
Doctorate degree: MD & PhD	1
Job/role	
Research assistant or coordinator	7
Research nurse	1
Genetic counselor	1
Geneticist	1
Trained genetic counselor	
Yes	1
No	9
Number of consenters at each site	
Site 1	2
Site 2	2
Site 3	2
Site 4	4
**Participants (total** ***n*** ** = 35)**	
Participant type	
Adult with a rare disease	14
Parent of a child with a rare disease	12
Parent of an adult with a rare disease	5
Other relative^a^ of an adult with a rare disease	4
Gender	
Female	16
Male	19
Ethnicity	
White British/white European	29
Asian	2
Algerian	1
Afghan	1
Black African	1
White British & Asian	1
Age	
Range 24 to 70 years, median 46 years	
**Appointments (total** ***n*** ** = 21)**	
Number of participants present	
One adult patient	10
Two parents of a child patient	6
One adult patient and one or more parents/relatives	5

^a^For example, a sibling

### Consent appointments: structure

**Fig. 1:**
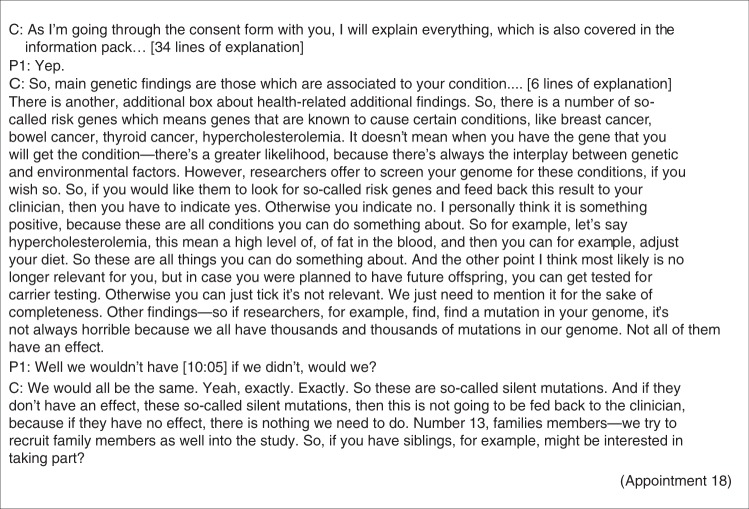
Fragment illustrating a consenter-led conversation where the participant was not actively involved in the conversation.

The appointments ranged from 11 to 52 minutes (median 27 minutes). Most (20/21) appointments were consenter-led, i.e., consenters led conversations using a structured approach in which they provided potential participants with information following the consent form order. Participants actively participated in the majority (14/20) of these consenter-led discussions, i.e., participants were involved in the conversation, expressing their views and/or any concerns. In the other six consenter-led appointments, however, there was little to no participant contribution and very little evidence of participants’ views (Fig. 1). One of these appointments may have been influenced by the child patient being increasingly agitated, shouting and screaming, requiring their parents’ attention. One appointment (A13) was participant-led: the participant deferred his decision; the conversation only covered a small part of the consent form. This was the shortest recording (11 minutes).

### Consent form topics covered by consenters

Almost all (20/21) consenters verbally covered the following topics from the written consent form: how personal health data will be accessed and used, confidentiality, main findings, participants can withdraw any time. Consent form topics covering uncertainty about disease risks associated with secondary findings (13/21), and how participants can change their minds about secondary findings via opt-in or opt-out forms (8/21), were covered less frequently (Table 2).

**Table 2:** Frequency with which 100,000 Genomes Project consent form topics were included in the discussion

Topic	Frequency topic included in discussion
What personal health data will be accessed and how it will be used	20
All information will be confidential	20
Information about main findings (including potential benefit to patient)	20
Participant can withdraw any time	20
Participant can choose whether they want to receive secondary findings	19
Information generated may have implications for participant's family members	19
Checking the participant has read the PIS and/or had opportunity to ask questions about PIS content	17
Information about giving blood and future samples	17
Commercial companies can access the participant’s data	17
The participant may be contacted by the clinical or project team in the future	17
Results may not be able to provide a diagnosis or change the participant’s care	17
Results may not be returned in a time frame that is clinically useful	17
How the participant's samples will be used	16
The participant will not benefit financially	16
Other findings (outside of agreed secondary findings) will not be routinely returned	16
Participation in the 100,000 Genomes Project is voluntary	15
Declining to take part in the 100,000 Genomes Project won’t affect the care the participant receives	13
Uncertainty about disease risks associated with secondary findings	13
Participant can change mind about secondary findings (through opt-in and opt-out forms)	8

Total *n* = 21. The consent form sections relating to “carrier status findings” are not reported here as these were only relevant to a subset of six appointments.

*PIS* patient information sheet

### Consenters’ uses of closed-ended versus open-ended questions

Consenters used closed-ended questions (e.g., “Any questions?”) to elicit participants’ questions in 12/21 appointments (totaling 30 instances). Although this sometimes (5/30) prompted participants to ask questions, most often (25/30) participants answered “No.” Consenters also used passive statements to invite participants' questions (e.g., “If you have any questions just ask”) in 13/21 appointments (totaling 14 instances): these rarely (1/14) elicited an immediate question. An open-ended question to elicit a participant’s questions, concerns, or views was used once by one consenter (“How does that sound?”) and this effectively elicited the view of the participant, who responded, “That sounds fine to me really, I’m quite happy for the broader screening as well. I mean I don’t see that, you know, is there, you know, it’s any help in the larger picture then I’m quite happy, because as you say I’m not aware of any other possible condition, but as you say in life certain things are not, you know, certainly children that’s not, no longer an issue for me” (A15).

### Consenters’ discussion of secondary findings

In 20/21 appointments, consenters provided brief information about secondary findings. Consenters often (20/21) used cancer as an example of potential secondary findings, specifically breast (15/21), colorectal/bowel (11/21), ovarian (5/21), prostate (3/21), and thyroid (3/21). “Some endocrine cancers,” “some male cancers,” and “Lynch syndrome” were referred to once each. Consenters referred to heart disease or hypercholesterolemia in 11/21 appointments (“a familial type of high cholesterol,” “some types of high cholesterol,” “some heart conditions,” “a few heart conditions,” “hypercholesterolemia”).

**Fig. 2:**
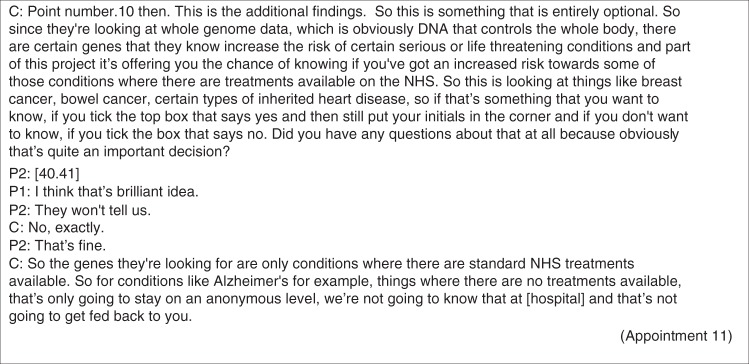
Fragment illustrating a consenter inviting participants’ questions specifically about secondary findings.

In most appointments (19/21), consenters were nondirective and emphasized secondary findings were optional. In two of these, consenters explicitly asked participants if they had any questions (Fig. 2); in three the consenters deliberately attempted to engage patients in the conversation (e.g., “How does that sound?” [A15]). In one appointment, the consenter expressed a personal opinion about secondary findings, i.e., was directive: “I personally think it is something positive, because these are all conditions you can do something about” (A18). In most appointments (16/21), consenters did not actively involve participants in conversation about secondary findings, e.g., in Fig. 1, the consenter talked through the consent form without pausing or asking the potential participant about their views regarding secondary findings.

### Participants’ questions

Participants in all except one appointment (A20) asked at least one question. Participants’ questions (109 total) fell into two broad areas with two-thirds (75/109) categorized as “substantive” and one-third (34/109) “administrative.” Substantive questions fell into three broad themes, described below.

#### Questions about research project details

Questions about research project details were raised in 13/21 appointments. First, participants had questions relating to practical/procedural aspects of the project, particularly relating to relatives. Specifically, some parents of children enrolling wanted to know what would happen regarding their child consenting and deciding about secondary findings when they turned 16 years old. Others asked whether and how other relatives could participate. Second, participants asked questions reflecting concerns about data access and security including questions about insurance or private health care, anonymization/confidentiality, future recontact about other research, and whether they could participate if they did not want commercial companies accessing their data. Third, patients asked questions indicating general interest about the project, including numbers of people recruited, whether this is a new project, and how long the research will continue. See Table S1 for illustrative quotes.

#### Questions about return of personal results

Participants asked about personal results in 14/21 appointments: the largest number of these related to secondary findings (9/21 appointments); fewer to main findings (4/21 appointments) or carrier findings (1/6 relevant appointments).

##### Questions about main findings

In two appointments, participants asked whether main findings would explain their child’s condition or inheritance type. One patient asked how long it would be until they received results. Another patient expressed concern about communicating main findings to his daughters, asking whether he could take part without receiving personal results. In another appointment, participants asked whether their main findings would be shared with their general practitioner (GP). See Table S1 for illustrative quotes.

##### Questions about health-related secondary findings

Participants asked about health-related secondary findings in 9/21 appointments, some to check whether they would receive secondary findings and others about the scope, especially relating to cancer. Two asked clarifying questions about their children’s secondary findings: one asked what “childhood onset conditions” meant; another asked for clarification about what would happen when their child turned 16.

##### Questions about carrier secondary findings

Carrier secondary findings were potentially relevant in six appointments; only one participant asked a question.

#### Questions about genetics and inheritance

In 8/21 appointments, participants asked about genetics and inheritance. Sometimes (4/21) these were questions to understand the inheritance patterns for the condition in their family. Others (3/21) asked questions about general genetics, specifically how many genes humans have, and how many genes or whether mitochondrial DNA might be involved in their condition. In two appointments, participants asked questions about the sequencing technology.

#### Administrative questions

One-third of participants’ questions (34/109) were practical questions about filling in the consent form, including what to fill in, which box to tick, who “the patient” and “relations” are.

### Participants’ discussion of health-related secondary findings

Themes arising when participants talked about secondary findings fell into four categories, described below.

#### Understanding of health-related secondary findings

In 13/21 appointments, there was insufficient evidence to make a judgment about whether participants understood the information about secondary findings. In the 8/21 appointments where participants did show some understanding, this was largely limited to understanding that only clinically actionable findings would be returned (e.g., “Yeah, if it’s something you can’t treat you won’t tell us?”) (see Table S2).

#### Positive attitudes toward health-related secondary findings

In nine appointments, participants expressed generally positive views regarding health-related secondary findings, e.g., “useful to know” (A10), “brilliant idea” (A11), “quite happy” (A15). Two participants alluded to this being empowering. One of these thought the information was “harmless” (A8), another that it was a novel opportunity (A3).

#### Negative attitudes toward health-related secondary findings

In two appointments, both including two parents, one parent expressed less positive views toward health-related secondary findings. In the first, the parent expressed concern about potential adverse psychological impact, e.g., “Yeah, no but that was what I was meaning, I don’t really want to be going ‘oh, you might have this,' great I’m going to worry about it for the rest of my life” (A2). In the second, the parent expressed uncertainty, e.g., “I’m not sure, I’m not sure for me, you can do what you want but…” (A3) (Table S2).

#### Participant-to-participant conversation about health-related secondary findings

In both cases where one parent expressed a negative/uncertain attitude about health-related secondary findings, this parent was convinced by the other parent to opt in. In two other appointments, participant-to-participant conversation about health-related secondary findings also occurred: these involved one participant simply telling the other to opt in (Table S2).

### Participants’ discussion of carrier secondary findings

Carrier status information was only relevant in six appointments: couples talked very little about this. Parents expressed positive views of the carrier secondary findings in 4/6 appointments but not in great depth, e.g., one just said they were “quite happy to” (A6). No negative attitudes toward carrier status information were expressed. In two appointments, parents evidenced some understanding of what being a carrier meant; in both cases, this was elicited by the consenter asking whether they understood what “carrier” meant.

## Discussion

Implementation of genome sequencing in mainstream clinical practice will require clinicians who are not genetics experts to discuss genome sequencing with patients. In this study we gained valuable insights into genome sequencing consent conversations between health-care professionals and patients. Broadly consistent with clinical research good practice guidelines, most discussions were consenter-led with active participant participation: consenters covered many information sheet topics, provided opportunities for questions, and participants expressed their views and/or concerns. The consenter-led approach increases the likelihood that essential information is communicated and is consistent with good clinical practice guidelines, as long as patients are actively encouraged to be part of the conversation.^10,16,17^

Having the opportunity to ask questions is key to informed consent,^18^ but in six appointments patients had little opportunity to express their concerns, views, or questions. Previous nongenetics research has similarly found consent conversations are often recruiter-led, e.g., in a cancer treatment consent study, 12 of 23 appointments were recruiter-led.^10^ In clinical practice, engaging patients in conversations and eliciting their questions, views, and evidence of understanding are well-established goals for patient–clinician communication.^19^ In genomics, as in other health-care areas, providers’ training may need to focus more on these aspects of consent. We found at least one appointment was often interrupted by a distressed child, perhaps reflecting particular challenges providers face when discussing consent in pediatric specialties.

Health-related secondary findings may be returned to 1 in 100 individuals having genome sequencing,^11^ and participants could opt in to the principle of receiving clinically actionable secondary findings (as against the gene list, which may change over time). Participants’ questions were often about secondary findings. Although most consenters told participants they could choose to receive secondary findings, fewer gave other important information. For example, a minority discussed the considerable uncertainty around secondary findings (e.g., what a *BRCA1* pathogenic variant would mean in the absence of a personal/family history of cancer), or that participants could opt in or opt out if they later changed their minds. Additionally, some relatives appeared to instruct others what to do regarding secondary findings. This emphasizes the need for health professionals to engage with all family members when discussing secondary findings, to ensure all make informed decisions reflecting their own values. Offering secondary findings will fall outside many health professionals’ expertise, posing a challenge if secondary findings are similarly offered to patients clinically, e.g., in the newly commissioned UK NHS genomic medicine service.^20^ Time is often pressured in clinical practice, but it is arguably just as important that patients have adequate information about secondary findings as about main findings. Health-care professionals may need to give more time to discussing secondary findings during genome sequencing consent conversations, and training for this discussion is a priority for future education strategies.

Participants less often asked questions about main findings, perhaps reflecting greater familiarity with the main diagnostic purpose of genome sequencing compared with the relatively novel considerations regarding secondary findings. Participants did ask questions about genetics and inheritance. This emphasizes the need for the health-care workforce to have sufficient knowledge to answer such questions or at least know where to direct patients if they cannot answer themselves. The 100,000 Genomes Project included investment in workforce development, recognizing the need to provide appropriate genomics education and training across health-care systems.^21^

Qualitative analysis of audio recordings provided insights into real-world consent, unlike interviews or focus groups, and allowed for in-depth exploration not possible in larger quantitative studies. Nonetheless, the small sample size limited generalizability. This methodology meant we could not explore participants’ knowledge or attitudes beyond what came up naturally in conversations. Additionally, we couldn’t evaluate whether participants made informed choices. Because we used audiotapes, body language and other signs of understanding could not be assessed, e.g., head nodding.

Few previous studies have directly explored communication around genome sequencing consent. Some have explored attitudes regarding next-generation sequencing consent using focus groups,^22,23^ interviews,^23–25^ and surveys^26^ conducted with participants (mostly parents)^22,23,25,26^ and health professionals.^22,27–29^ These shed some light on stakeholders’ sequencing attitudes and information needs. For example, in a mixed-methods study of 25 parents, desire for secondary findings was high.^23^ In a survey of 760 health professionals, many thought secondary findings should be offered.^29^ Consistent with our study, previous focus group research found parents were cognizant of risks such as anxiety about secondary findings.^22^ Our study builds on previous studies by examining the actual consent process, allowing us to shed light on what questions patients actually ask of health professionals when given the opportunity, and how health professionals actually communicate with patients about main and secondary findings.

Our findings highlight issues that need addressing in health professional training to support genome sequencing consent processes and implementation. All consenters in the 100,000 Genomes Project underwent 1.5 hours of standardized online training prior to consenting potential participants, additional local training and supervision, and continued professional support.^12^ Such training undoubtedly is critical in the future scaling-up of clinical genome sequencing. As the 100,000 Genomes Project is completed, genome sequencing is being integrated into UK NHS clinical practice for selected indications. Nongenetics health-care providers need to be able to talk with patients about next-generation sequencing–based tests including genome sequencing. Our findings have implications for consent as genome sequencing transitions from research to clinical implementation.

Based on our study findings, we recommend standardized training about next-generation sequencing consent include greater emphasis on how to engage patients in the conversation. In the 100,000 Genomes Project consent training, consenters are informed they should “listen,” give patients “time to think,” engage in “dialogue not monologue,” and support patients in making informed decisions. However, little guidance is given on how to do these things. Health professionals will need training in more concrete communication and listening techniques to achieve these goals, e.g., the well-established counseling techniques of active listening, open-ended questions, and ceding the floor^30–32^ (see Box 1). Consenters exhibiting these behaviors were more likely to elicit participants’ views, concerns, and questions. A basic checklist for health-care providers including guidance such as “Use open-ended questions to elicit patients’ views, concerns, and questions” could help ensure patients at minimum have opportunities to participate and ask questions. Helping participants/patients make informed choices regarding secondary findings, e.g., discussing the surrounding uncertainty and that they can opt in or opt out later on, also needs greater attention in future education.

A final consideration is that the study was conducted in a novel hybrid clinical–research setting with dedicated consenters. As Wade et al. emphasize, in research, “an inherent tension exists between safeguarding informed decision-making by participants and maximising numbers enrolled.”^10^ The 100,000 Genomes Project differs from traditional research and future clinical settings where consent conversations will happen during routine clinical care. However, certainly in clinical settings in England, future patients will be invited to consent to their sequence and other data being used for research as well as personal clinical purposes. Our study therefore provides insights on genome sequencing consent that are relevant to future clinical contexts. We conclude that future health professional training and/or communication/decision aids about genome sequencing may valuably focus on secondary findings and skills to increase patients’ active involvement in the consent process.

Box 1 Recommendations for health-care professionals discussing genome sequencing with patients as part of the informed consent process

## Electronic supplementary material

Supplemental Tables

Supplemental Information
