# Supplementary material for: Opening the “black box” of informed consent appointments for genome sequencing: a multisite observational study
Source: Genet Med. 2018 Oct 1;21(5):1083–91. doi: 10.1038/s41436-018-0310-3 (PMC6752270; doi:10.1038/s41436-018-0310-3)
Supplement: Supplementary file 1 — Supplemental Tables [file 41436_2018_310_MOESM1_ESM.pdf]

**Supplemental Table 1. Participants' questions: themes, sub-themes and illustrative quotes**

| 'Substantive' Themes / Sub-themes | | Illustrative quotes |
|---|---|---|
| **1. Questions about research project details** | | |
| **1.1. Questions relating to practical / procedural aspects of the project** | **Participant consenting at 16** (Child re-consenting at 16 / Determining capacity at 16) | *P1 I mean, how does it happen that he hasn't got the capacity to decide, does it go through the court or some sort of –* (Appointment 1)<br><br>*P1 If when she's 16, her – she's not able to say herself, what's the situation then?* (Appointment 5) |
| | **Family involvement / relevance to the project** Eligibility of other relatives / reasons for not sequencing child's siblings / Relatives taking part in project remotely / logistics of consent | *P1* ***And what about because obviously we have a son as well but they didn't ask screening with a sibling?***<br>*HP We only take samples from siblings if they're either affected with the same condition or there's a query that they might be affected -*<br>*P1 Oh OK, yeah, no there isn't at all. I just wondered if you would, sort of, whether it would help you to see, obviously he's a boy, she's a girl, so straightaway there are going to be differences -*<br>*HP Yeah.*<br>*P1 - but I didn't know whether for you guys doing the research whether something would jump out that he hadn't got that she had got or, you know –* (Appointment 6)<br><br>*P1 -* ***I have two other siblings, you know, would that be of interest?***<br>*HP We can get one more sibling so for every patient we can take up to two unaffected siblings.*<br>*…*<br>*HP Yeah, OK. So if I leave you with a copy of the leaflet to pass on to her and if she is interested in taking part, if you get her to contact me either by email or phone, my details are on the back of the leaflet.*<br>*P1 Mm, OK.*<br>*P2* ***So really all she would need do is signing like that consent form, would she have to then go to a doctors close by for the blood test?***<br>*HP Yeah, exactly. A doctor nearby to her and we would send all of that out in the post to her, so she would take the tubes and everything along to the GP surgery and then get that posted back. I mean, that's only if she's happy to do it -*<br>*P2 Yeah, I think she may well be, yeah. (Appointment 10)* |
| **1.2. Questions reflecting concerns about data access and security** | **Insurance / private healthcare** | *HP Currently you don't ask to have insurance companies that are taking part in genetic research. I can't guarantee that will never change.*<br>*P1* ***If they were to be – you know, if they had to be told, then what difference would that make if at all?***<br>*HP It could have an effect both, depending on what [0:11:02 child shouts obscuring HP voice] positive and negative.* (Appointment 5) |

| | |
|---|---|
| | *P2* ***This is a really, probably a silly question but I just need to ask it, doing this won't negate any private health care, things will it.***<br>*HP No.*<br>*P2 So say I develop the disease and I'm, it doesn't make it like a known condition it's not known.*<br>*HP No.*<br>*P4 Otherwise all the private insurances would [15:30] every time, so they could turn round and say you're never going to break it so they won't.* (<u>Appointment 7</u>) |
| **Anonymisation / confidentiality** | *HP So what you're signing to point 4 is to say that you're happy for your anonymised health records to be stored alongside your samples and used in order to help interpret your samples too.*<br>*P1* ***So in order to get them they will obviously have to use my name wouldn't they but then they would remove the name afterwards.***<br>*HP Yes, so all the anonymisation would be done here, so we'd gather the information that was requested, we'd take away any personal identifying information and then we'd submit that to the central database.* (<u>Appointment 9</u>) |
| **Future re-contact about <u>other</u> research projects** | *HP All of those studies, if they contact you, they're all voluntary at the time, just because you say yes to this one does not mean you have to agree to -*<br>*P1* ***Do they normally contact, this is a really stupid question, but do they normally contact you by post or email or -***<br>*HP It would probably be post because it would probably -*<br>*P1* ***Yes, that's OK, because you know how many terrible phone calls you get and you slam the phone down -***<br>*HP No it's more likely that it would be through post or it would come from us and-*<br>*P1 Yeah, passing it on, yeah that's fine.* (<u>Appointment 6</u>) |
| **Can I take part and NOT have commercial companies access my data?** | *P2 So my name here [17.05] –*<br>*HP Yes but only if you're happy with the commercial companies.*<br>*P2* ***Oh yeah, it's a sticking point for me, this commercial company thing, I'm not too sure, to be honest –***<br>*P1* ***So if he doesn't want to then he will not have his tests done?***<br>*HP Yeah.*<br>*…*<br>*P2 So you're saying I can't take part on this?*<br>*HP No, unfortunately not. It is a sticking point for quite a lot of people so you're not alone on this one.*(<u>Appointment 1</u>) |
| **Financial cost to patient** | *P1* ***Just I need to ask: there is no cost in this at all? It's just me…?***<br>*HP Pardon?*<br>*P1* ***There's no cost for me? It's just a—?***<br>*HP Oh, no. No.*<br>*P1 That's fine.*<br>*HP No, no.*<br>*P1 Just need to ask.*<br>*HP Yeah, that's—*<br>*P1 I always feel you have to ask that. (<u>Appointment 16</u>)* |

| | | |
|---|---|---|
| **1.3. Questions indicating general interest about the project** | **Numbers of people recruited into the project** | *HP2: OK, just make a fist for me please. Good.*<br>*P1:* ***So how many volunteers has the project got so far do you know?***<br>(Appointment 10) |
| | **Is this a new project** | *HP Yeah. You're welcome. So the number four is the use of your health data, which is already – we have so far for you in our system.*<br>*P1* ***Is this a very new project?***<br>*HP This one – this actually, um…we started enrolling from this particular hospital September of 2015, but this was endorsed by David Cameron 2012.*<br>(Appointment 16) |
| | **How long is the research going to continue (inc. withdrawal)?** | *P1* ***And how long will the information from the study be available for researchers, you know, time wise, years?***<br>*HP Indefinitely.*<br>*P1 Indefinitely.*<br>*HP Unless you withdraw from the project.*<br>(Appointment 11) |
| **2. Questions about return of personal results** | | |
| **2.1. Questions about main findings** | **Will the main findings be able to tell us the cause / inheritance type?** | *HP We will need to confirm that this is a recessive inheritance again based on the family history it's likely to be recessive but we will need to confirm this first.*<br>*P3* ***And will this study do that just to be clear?***<br>*HP Yes, so if we can identify the gene then we will be able to confirm the inheritance pattern*<br>(Appointment 7)<br><br>*P3* ***So [2.00] explain his behaviour?***<br>*HP It might do, it depends, obviously there are some kind of genes that we know [] different conditions, some of them behavioural, some are to do with [] of development, I can't guarantee we're going to be able to give you an answer as to what the eventual cause is.*<br>(Appointment 4) |
| | **Turn-around time for results** | *HP If we find them we'll tell you them. Then you get the opt in to the additional health findings -*<br>*P1:* ***And like is all that held in that 18 months, so it's not - ?***<br>*HP It might take a lot longer than that, it depends on how things develop.*<br>(Appointment 3) |
| | **Concern about communication of results with family members (or lack of)** | *P1 I mean is that, presumably there's a point in using the samples and stuff subject to medical research. It doesn't really matter,* ***it's no reflection on to the research if I chose not to tell my daughters?***<br>*(Appointment 13)* |
| | **Sharing of personal health information e.g. with GP** | *HP Do you have questions after reading that at all?*<br>*P2 [1.27] to get a copy of anything that's found?*<br>*HP So they would, yes. So initially our patients' GPs get a letter to confirm that* |

| | | |
|---|---|---|
| | | *they're part of the study but the relatives don't routinely get that, but when the report comes in at the end, which will have the information relating to your son's eye condition plus if you've opted in to any of the additional findings, it might show that you have certain risks of other conditions and your GP would get a copy of that, yes.*<br>*…*<br>*P2* ***How do you know who my GP is?***<br>(Appointment 11) |
| | **Can I take part in the research without receiving any personal results?** | *P2* ***It could say give you the blood sample and you do all your tests and not have the result, but it might be useful to you?***<br>*…*<br>*P2 Yeah no I was just thinking if you didn't want to know, but yet your blood sample would still be useful then, you know, that's fine but yeah.*<br>(Appointment 13) |
| **2.2. Questions about health-related secondary findings** | **What additional health-related findings will I / we be informed of? (scope)** | *HP With stuff like Alzheimer's disease or [0:17:18]*<br>*P1 Yeah, exactly*<br>*P2* ***Yeah, if it's something you can't treat you won't tell us?***<br>*HP We won't tell you about.*<br>(Appointment 2)<br><br>*HP So I don't know if anyone mentioned as well that because we are looking at all of the genes, you do get the option of finding out certain other information too?*<br>*P1* ***Yeah, I was going to ask about that. If they picked up anything else would they let me know if I've inherited anything else?***<br>*HP There are certain things they would, it's entirely up to you, so there's an option in the consent form where you can pick yes or no -*<br>*P1 Right.*<br>*HP - there I think between ten and twelve conditions at the moment that they look for, that can be things like breast cancer, bowel cancer, other sort of serious life threatening conditions but where there are treatments available on the NHS.*<br>*P1* ***Would it be able to pick up whether like in the future that I've got a higher risk of maybe say having heart disease or something like that?***<br>*HP So I think certain types of heart disease and conditions would be covered but not on a more general basis, so they're looking for conditions where there's one particular gene that massively increases your risk, so not sort of a gene that would increase it by half a percent or something.*<br>*P1 No.*<br>*HP And also not conditions where there are sort of dozens of different genes involved, like with breast cancer for example we know of a couple of different genes and if you have the set changes in either of those genes, your likelihood of developing breast cancer increases quite significantly.*<br>*P1 OK.*<br>(Appointment 9)<br><br>*P1* ***If they did find something else I suppose if there was a follow up, yeah that's I mean -*** |

| | |
|---|---|
| | *HP Yeah so you'll be contacted.*<br>*P1 Yeah if it was found they'd -*<br>*HP Definitely.*<br>*P1 They would, ok.*<br>(Appointment 15)<br><br>*HP No.10 is the optional one that I mentioned earlier, so the predictive testing, so again the things that we would look for here would be to look for if you're a higher risk of developing breast cancer, colorectal cancer, ovarian cancer, prostate cancer –*<br>*P1 Will that be tested?*<br>*HP If you choose for it, so you can tick yes or no for this, if you tick yes then they'll feed that back to you. If you tick no then they won't look for it.*<br>*P1 Oh so they will look for those cancers?*<br>*HP Yes, so if you're at a higher risk.*<br>*P1 In this sample, if I tick yes that's what they're going to look, just check?*<br>*HP Yeah.*<br>*P1 OK.*<br>(Appointment 1) |
| **2.3. Questions about carrier secondary findings** | *HP And the second thing we could do as a couple is we can look at carrier status. Do you know what it means to be a genetic carrier of a condition?*<br>***P1 Is that where you can have the genes that you don't necessarily -?***<br>*HP So because you've got, everyone has two copies of every gene, you can carry an observational one that means that that particular copy doesn't work or that because you've got a second one you're healthy. But if two parents with changes in the same gene have children there is a risk that the child can have all [0:05:18] copies and develop a condition.*<br>*P1 Right, yeah, yeah.*<br>(Appointment 2) |
| **3. Questions about genetics and inheritance** | |
| **3.1. Questions to understand the inheritance patterns for the condition in their family (recessive vs dominant inheritance)** | HP Yes. So with x linked conditions they can't go from men to men. It can go from females to everyone, it can go from men to women but not father to a son.<br>P2 **So it should end with him?**<br>HP Unless your daughter was a carrier.<br>(Appointment 11)<br><br>P1 **I mean there's always, I mean if it's recessive – I understand the set of genes to do, it could skip a generation couldn't it?**<br>(Appointment 13)<br><br>P3 **Do you personally have a lot of contact with people with this condition or not?**<br>HP Yes, yes.<br>(Appointment 7) |

| | | |
|---|---|---|
| **3.2. Questions about general genetics** | **How many genes do we have?** | P1 **How many genes we've got, 20,000?**<br>(Appointment 9) |
| | **Mitochondrial DNA** | P4 [22:45] **whether there's any Mitochondrial DNA connection at all?**<br>HP Um.<br>P4 Because that's slightly different now isn't it?<br>HP Yes, it could happen but it's always.<br>…<br>P4 **So they haven't thought that there's any Mitochondrial DNA connections?**<br>HP Um, no.<br>(Appointment 7) |
| **3.3. Questions about the sequencing technology** | **How is the DNA stored (frozen)?** | *P2* ***How do you store, I mean, is it frozen or - ?***<br>*HP Once the DNA's been extracted, so that happens today, that actually gets stored at room temperature in a fridge, so it can then be stored -*<br>*P2 Forever.*<br>*HP Forever, yeah.*<br>*P2 Wow.*<br>(Appointment 4) |
| | **How is the DNA extracted?** | P1 **How do actually extract the DNA because -**<br>HP Spin it really fast... (continues…)<br>(Appointment 4) |

**Supplemental Table 2.** **<u>Secondary findings</u> – themes, sub-themes and illustrative quotes**

| ‘Substantial’ Themes / Sub-themes | Illustrative quotes |
|---|---|
| **1. Understanding of secondary health-related findings** | |
| **1. Scope / types of results returned (only clinically actionable)** | *HP Yeah, so we won’t tell you anything else, any other findings that we get unless there’s something –*<br>***P1 Yeah, just the ones we’ve discussed, OK.***<br>*HP Yeah, just the ones that are related to [Name] and anything that kind of falls into the additional findings categories that have to, they have to be serious conditions, but they have to be something that we can do something about.*<br>***P1 Treat them, yeah, yeah.***<br>***P2 Right, OK, so you won’t just tell us –***<br>***P1 No, but that’s what I didn’t want to know, that’s what I mean.***<br>***P2 Yeah, but***<br>***[all talking together]***<br>*HP With stuff like Alzheimer’s disease or [0:17:18]*<br>***P1 Yeah, exactly***<br>***P2 Yeah, if it’s something you can’t treat you won’t tell us?***<br>*HP We won’t tell you about.*<br>(Appointment 2) |
| **2. Positive attitudes towards health-related secondary findings** | |
| **2.1. Generally positive views** | *“useful to know”* (Appointment 10) |
| **2.2. Empowerment** | *HP And number ten, so this is the additional medical findings, so these are the ones related to the cancer genes and the high cholesterol genes which is what we’re looking at currently.*<br>*P1 Right, OK.*<br>*HP So this is optional and you can change your mind.*<br>*P1 Yeah.*<br>*HP So if you want, I need to put an option today on the consent form [0:15:35]*<br>*P1 Yeah, yeah.*<br>*HP But you can change your mind.*<br>*P1 I think you’re going to do it and if we’re going to do it for [Name] then I might as well do it and cover ourselves this week. Because if something came back with you I’d be worried ‘oh, what’s it going to say’, but I’ll do it because we might as well all do it together.*<br>*P2 Well, yeah.*<br>*P1 We can worry about the results afterwards.*<br>***P2 Yeah, forewarned is forearmed surely.***<br>*P1 OK.*<br>(Appointment 2) |
| **2.3. Harmless** | *HP: So the other um, significant point that [Name 1] probably went through in clinic is that in addition to finding out about your eye condition, um you will get the option of finding out some other bits of information as well… (several lines of explanation…)* |

| | |
|---|---|
| | P: ***It doesn't bother me you can do it, it's harmless if anything it's beneficial to know init, so.***<br>*HP: I mean yeah, there are certainly certain benefits that could, I mean you can get screening earlier for example if you know that there are risks, you can sort of know what to look out for. Some people choose not to find out because their worried about um, on an emotional level perhaps… (several lines of explanation).*<br>***P: I'm ok with it.***<br>(Appointment 8) |
| **2.4. Novel opportunity** | *HP So will you want it?*<br>*P1 Yeah, no that's fine, you can do the additional as well.*<br>*HP The only ones we'll tell you about are ones that are serious that we can do something about within the Healthcare system.  So we can either screening earlier for cancers and that kind of thing.*<br>***P2 Yeah, yeah, yeah, yeah.***<br>*HP And looking at different kinds of types of medication.*<br>***P2 Well, that is actually, that's a bit of detail that you can't – because you're going to get access to something that the general public don't generally get to look at.  So for you to turn that up is a very serious like –***<br>*P1 No, that's fine, I agree with you.*<br>***P2 You're better to know because preventing it maybe it will save you long term if you've got a chance to do something.***<br>*P1 Yeah, no that's fine, yeah, OK, we'll do it all.*<br>(Appointment 3) |
| **3. Negative attitudes** | |
| **3.1. Concern about potential adverse psychological impact of results** | ***P1 No, the only thing that I said to them on the phone, because it was suggested to us to do this, was that I just, this was really funny, no, it isn't that relevant, I just want to look at the one area because I don't want to open up a can of worms.***<br>*HP OK, that's fine.*<br>***P1 I just want to look at the cardiac side of things don't you think?***<br>*P2 Yeah.*<br>***P1 But I don't yeah, that's what I basically said to that.***<br>*HP Yeah, so I will do what you can back here, if you don't want the initial findings then we won't give them to you.  We won't give you information you don't want, obviously.*<br>***P1 Yeah.***<br>*…*<br>*HP Yeah, just the ones that are related to [Name] and anything that kind of falls into the additional findings categories that have to, they have to be serious conditions, but they have to be something that we can do something about.*<br>*P1 Treat them, yeah, yeah.*<br>*P2 Right, OK, so you won't just tell us –*<br>***P1 No, but that's what I didn't want to know, that's what I mean.***<br>*P2 Yeah, but*<br>*[all talking together]*<br>*HP With stuff like Alzheimer's disease or [0:17:18]*<br>***P1 Yeah, exactly***<br>*P2 Yeah, if it's something you can't treat you won't tell us?*<br>*HP We won't tell you about.*<br>***P1 Yeah, no but that was what I was meaning, I don't really want to be going 'oh, you might have this, great I'm going to worry about it for the rest of my life'.***<br>*HP Yeah, that was definitely what we're trying to avoid is causing people unnecessary –* |

| | |
|---|---|
| | ***P1 Yeah, no that's understandable.***<br>*HP Worry.*<br>(Appointment 2) |
| **3.2. Uncertainty about the decision** | *HP And that's, it's optional, you don't have to have this information.*<br>*P2 No, no I would like to have that information, yeah it would give us, that –*<br>***P1 I'm not sure, I'm not sure for me, you can do what you want but –***<br>*P2 No, but that's up to you, that will give me an insight and understanding of any potential risks, so I would rather know so that you would be able to [0:06:19]*<br>***P1 Yeah, no, yeah I agree.***<br>(Appointment 3) |
| **4. Participant-to-participant conversation about health-related secondary findings** | |
| **4.1. One participant convinces other participant to opt in** | *HP The only ones we'll tell you about are ones that are serious that we can do something about within the Healthcare system. So we can either screening earlier for cancers and that kind of thing.*<br>*P2 Yeah, yeah, yeah, yeah.*<br>*HP And looking at different kinds of types of medication.*<br>*P2 Well, that is actually, that's a bit of detail that you can't – because you're going to get access to something that the general public don't generally get to look at. So for you to turn that up is a very serious like –*<br>*P1 No, that's fine, I agree with you.*<br>***P2 You're better to know because preventing it maybe it will save you long term if you've got a chance to do something.***<br>***P1 Yeah, no that's fine, yeah, OK, we'll do it all.***<br>(Appointment 3) |
| **4.2. One participant tells other participant to opt in** | *HP For point 10 then, this is entirely optional, so we do need your initials in the corner regardless but then there is an option of yes or no and those boxes are over on the left hand side. So this is about whether or not you want to find out additional findings unrelated to your eye condition. So this is looking at whether or not you want to know if you're at risk of certain treatable conditions, so this is breast cancer, bowel cancer, other conditions where we know a specific gene that causes a condition where there's a treatment available on the NHS. So if that's some information that you want to know, if you tick the top box that says yes. If you don't want to know, if you tick the bottom box that says no.*<br>***P1 Tick yes.***<br>***P2 Tick yes?***<br>***P1 Yes.***<br>*HP So obviously with these results, have you both put yes for that?*<br>*P1/2 Yeah.*<br>(Appointment 10) |
