# Supplementary material for: Opening the “black box” of informed consent appointments for genome sequencing: a multisite observational study
Source: Genet Med. 2018 Oct 1;21(5):1083–91. doi: 10.1038/s41436-018-0310-3 (PMC6752270; doi:10.1038/s41436-018-0310-3)
Supplement: Supplementary file 2 — Supplemental Information [file 41436_2018_310_MOESM2_ESM.pdf]

100,000 Genomes Project

Hospital logo here

Participant consent form

If you agree to take part in the 100,000 Genomes Project, please *initial* every box, *tick* the appropriate option in boxes 10 and 11, and *sign* at the end of this form.

1 I have read and understood the participant information sheet 'For adults with a rare genetic disease' or 'For adult family members of patients with rare genetic diseases' dated ______________ (version _____) for the 100,000 Genomes Project. I have had the opportunity to ask questions and have had these answered satisfactorily.
I understand that my participation in the 100,000 Genomes Project is voluntary and that if I decline, I don't need to give any reason and that my present or future medical care or legal rights will not be affected.

Initial here to indicate your agreement ☐

2 **Sample collection**

I agree to give a sample of blood, and for samples already collected as part of my medical care to be used. I also agree to give other samples such as saliva if necessary.

I agree to being asked by my clinical team to provide further samples in the future for the purposes of the Project. I understand that agreeing to be asked for further samples does not mean that I have to provide them.

I agree that details about me and any samples I provide will be stored securely by Genomics England.

Initial here to indicate your agreement ☐

3 **Use of samples**

I agree that my donated sample(s) can be used to collect DNA for whole genome sequencing, and for studies looking at proteins or other components of my cells.

I understand that my samples or DNA could be sent to approved organisations outside the UK for secure processing or analysis.

I understand that future research on my samples may use new tests or techniques that are not yet known.

Initial here to indicate your agreement ☐

4 **Use of health data**

The 100,000 Genomes Project allows medical researchers, healthcare teams and commercial organisations to access samples and information collected by the Project.

I agree that the Project can access, collect, store and analyse information from my medical notes, health records and personal information (from my GP or hospital or social care records, or other sources such as local or national disease registries), to be used alongside my samples for scientific or medical purposes and research relating to medical condition(s) affecting me or other people.

I understand that this can be at any point during my life and will continue after my death, unless I have withdrawn from the Project.

I understand that researchers won't be allowed to copy or remove any of my information.

I agree that these notes and records or the samples I give may be looked at by approved individuals from Genomics England or from the NHS Trust and other study monitors at any time.

Initial here to indicate your agreement ☐

5 **Confidentiality**

I understand that all information about me held by the Project will be treated as confidential. I understand that information from my samples, records or other information I give to the Project will only be accessible to researchers other than my clinical team in a form that protects my identity.

I understand that my GP and other healthcare professionals may be informed of my participation in the Project.

Initial here to indicate your agreement ☐

6 **Access for commercial companies**: I understand that the research organisations accessing the data could include commercial (for-profit) companies.

Initial here to indicate your agreement ☐

7 **Financial implications:** I understand that I will not benefit financially if research undertaken through the 100,000 Genomes Project leads to new treatments or medical tests.

Initial here to indicate your agreement ☐

8 **Future contact**

I agree to be contacted by my **clinical team**, or directly by the **Project team** for more information about my health, or to be invited to participate in future research studies.

I understand that this research may be about this Project or other ethically approved research studies, including clinical trials or research about my experience of the Project.

I understand that I will be provided with full information about these studies when and if I am contacted, and that agreeing to be contacted does not mean that I have to take part.

Initial here to indicate your agreement ☐

9	**Main genetic findings *(agreement to this is necessary to take part in the Project)*:**

I give consent for the Project to run tests on my samples and health information relating to the cause or management of the main condition that was the reason I (or my relative) was invited to join the Project. (Main findings are also sometimes called 'primary' or 'pertinent' findings).

I agree that these results can be reported to my clinical team for them to discuss with me.

I understand that the results may **not** be able to provide a diagnosis, or to provide information to help with my clinical care (or the care of my relative).

I understand that the results may **not** be returned in a time frame which will allow them to be used in my clinical care (or the care of my relative).

Initial here to indicate your agreement ☐

10	**Health-related additional findings *(optional)*:**

I understand that I can choose whether or not I want the Project to **look in my genome data for additional genetic results, beyond my (or my relative's) main findings.** (Additional Findings are also sometimes called 'secondary' findings).

I understand that if I choose to receive these findings, the Project will provide me with findings that are likely to benefit me because I may be offered screening or treatment as a result. However, I understand that there is still uncertainty about such findings and I may not benefit from receiving them.

I understand that if no additional findings are found, I may still be at risk of the conditions they can cause.

I understand that I can change my mind about whether to receive these results at any time by completing an Opt-in or Opt-out form.

Tick your choice (✓)

☐ Yes, I want this information to be looked for and fed back to my clinical team

OR

☐ No, I do not want this information to be looked for and fed back to my clinical team

Initial here to confirm your choice ☐

11 Reproductive additional findings (carrier testing) *(optional)*

I understand that my partner/spouse and I can choose whether or not to be tested to see if we 'carry' a risk of passing on serious or possibly life-threatening genetic conditions to our future children. These conditions may or may not be able to be cured, made less severe, or prevented via standard NHS treatment.

I understand that most of these carrier test results are offered – and will be returned to us – together as a couple, only if both of us give consent to this. If we **don't** both consent together, this information will **not** be looked for.

I understand that medical conditions **that can only be passed on to children by one member of the participating couple** may be included. In this case, results will be given individually to that member of the couple by the clinical team. Both partners/ spouses do not need to consent together to have this testing.

I understand that one or both of us may still have a chance of having a child with one of the studied conditions, even if the additional findings analysis doesn't identify anything in me/us.

I understand that I can change my mind about whether to receive these results at any time by completing an Opt-in or Opt-out form.

Tick your choice (✓)

☐ **Yes, I want this information to be looked for and fed back to my clinical team**

OR

☐ **No, I do not want this information to be looked for and fed back to my clinical team**

OR

☐ **Carrier testing is not relevant for me**

Initial here to confirm your choice ☐

12 Other findings: I understand that any other results of genetic or other analysis of my samples (which are not related to my main condition or additional findings) will not routinely be fed back to me.

Initial here to indicate your agreement ☐

13 Family members: I understand that information generated by this Project may be of benefit to my family members now or in the future. I understand that the clinical team will advise and support me with sharing this information with my family members if this is the case.

Initial here to indicate your agreement ☐

14 **Withdrawing from the Project:** I understand that I am free to withdraw my permission for my samples and information to be used at any time in the future. I don't need to give a reason and my routine medical care will not be affected.

I understand that if I join and then withdraw from this Project it will not be possible to remove my data or samples from research that may already have taken place.

Initial here to indicate your agreement ☐

Name of patient (BLOCK CAPITALS)

Date of birth DD / MM / YY

Signature

Date DD / MM / YY

Name of person taking consent (BLOCK CAPITALS)

Signature

Date DD / MM / YY

Name of interpreter if used (BLOCK CAPITALS)

Signature

Date DD / MM / YY

When completed:

1 (original) to be kept in the adult participant's 100,000 Genomes Project records.
1 copy for participant.
1 copy to be sent to Genomics England.

INSERT LOCAL CONTACT DETAILS/
LABEL HERE
